# A screening system to identify transcription factors that induce binding site-directed DNA demethylation

**DOI:** 10.1186/s13072-017-0169-6

**Published:** 2017-12-08

**Authors:** Takahiro Suzuki, Shiori Maeda, Erina Furuhata, Yuri Shimizu, Hajime Nishimura, Mami Kishima, Harukazu Suzuki

**Affiliations:** 1 Division of Genomic Technologies, RIKEN Center for Life Science Technologies (CLST), RIKEN Yokohama Campus, 1-7-22 Suehiro-cho, Tsurumi-ku, Yokohama City, Kanagawa 230-0045 Japan; 2 Graduate School of Medical Life Science, Yokohama City University, 1-7-29 Suehiro-cho, Tsurumi-ku, Yokohama City, Kanagawa 230-0045 Japan

**Keywords:** DNA demethylation, Transcription factor, Binding site

## Abstract

**Background:**

DNA methylation is a fundamental epigenetic modification that is involved in many biological systems such as differentiation and disease. We and others recently showed that some transcription factors (TFs) are involved in the site-specific determination of DNA demethylation in a binding site-directed manner, although the reports of such TFs are limited.

**Results:**

Here, we develop a screening system to identify TFs that induce binding site-directed DNA methylation changes. The system involves the ectopic expression of target TFs in model cells followed by DNA methylome analysis and overrepresentation analysis of the corresponding TF binding motif at differentially methylated regions. It successfully identified binding site-directed demethylation of SPI1, which is known to promote DNA demethylation in a binding site-directed manner. We extended our screening system to 15 master TFs involved in cellular differentiation and identified eight novel binding site-directed DNA demethylation-inducing TFs (RUNX3, GATA2, CEBPB, MAFB, NR4A2, MYOD1, CEBPA, and TBX5). Gene ontology and tissue enrichment analysis revealed that these TFs demethylate genomic regions associated with corresponding biological roles. We also describe the characteristics of binding site-directed DNA demethylation induced by these TFs, including the targeting of highly methylated CpGs, local DNA demethylation, and the overlap of demethylated regions between TFs of the same family.

**Conclusions:**

Our results show the usefulness of the developed screening system for the identification of TFs that induce DNA demethylation in a site-directed manner.

**Electronic supplementary material:**

The online version of this article (10.1186/s13072-017-0169-6) contains supplementary material, which is available to authorized users.

## Background

DNA methylation of CpG dinucleotides at gene regulatory regions is a fundamental epigenetic modification for gene silencing. CpG dinucleotides are usually highly methylated, except at regulatory regions such as promoters and enhancers, where methylation levels tend to be anti-correlated with downstream gene expression [1]. During cell differentiation, DNA methylation acts as a safeguard to prevent the expression of unnecessary genes, while regulatory regions of master transcription factors (TFs) must be demethylated before or during differentiation [2]. Abnormal DNA demethylation is associated with several serious diseases such as cancers [3, 4], suggesting that the dynamics of DNA methylation are strictly regulated to establish cell type-specific gene expression during cellular differentiation.

De novo DNA methylation is regulated by the DNA methyltransferases DNMT3a and DNMT3b [5, 6]. Once the CpG cytosine is methylated by these enzymes, the methylated status is inherited by daughter cells during cell division via the maintenance DNA methyltransferase DNMT1 [7, 8]. Active demethylation involves further steps, including a series of oxidizations by ten-eleven translocation (TET) enzymes and a base excision repair pathway [9–13]. Demethylation can also be passive, in which methylated CpG is diluted upon DNA replication without DNMT1 maintenance of the methylated status.

While enzymatic mechanisms of DNA methylation and demethylation are well characterized, little is known about how DNA methylation dynamics are spatiotemporally regulated. Recently, some TFs such as SPI1 were shown to promote DNA demethylation in a binding site-dependent manner [14–17]. We also demonstrated that RUNX1 induces DNA demethylation by recruiting DNA demethylation machinery to its binding sites, which likely contributes to hematopoietic development [18]. These studies indicate that TF-mediated DNA demethylation plays an important role in gene regulation, but TFs that induce binding site-directed DNA methylation changes are poorly characterized.

Here, we develop a novel versatile screening system to identify TFs that induce DNA methylation change in a binding site-directed manner. Our system involves the ectopic expression of the target TF in model cells, subsequent methylome analysis, and overrepresentation analysis of the corresponding TF binding motif (TFBM). We also report the identification of novel binding site-directed DNA demethylation-inducing TFs using our system.

## Results

### Assessment of a novel approach to identify TFs that induce binding site-directed DNA demethylation

**Fig. 1:**
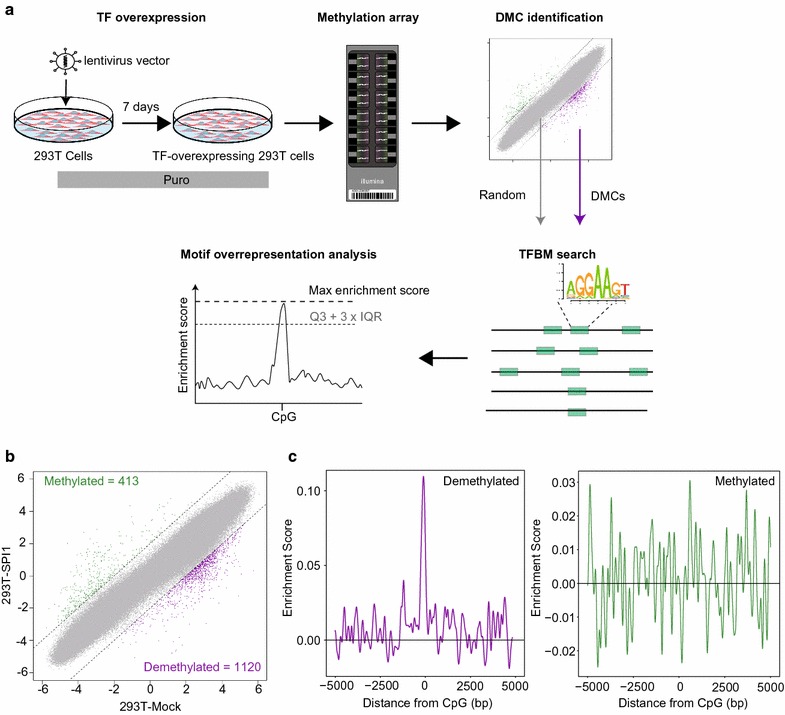
Feasibility of the approach to identify TFs that induce binding site-directed DNA methylation. **a** Flowchart of the approach. The TF of interest is overexpressed in a lentivirus vector that co-expresses a puromycin-resistant marker in 293T cells. Puromycin selection is applied for 7 days; then, TF-overexpressing 293T cells are subjected to DNA methylation analysis to identify differentially methylated CpGs (DMCs). Corresponding TFBMs of overexpressed TF are identified and then assessed to determine whether they are overrepresented at DMC regions. **b** Scatter plot showing DMCs caused by SPI1 overexpression in 293T cells. *X*- and *Y*-axes show *M*-values for 293T-mock and SPI1-overexpressing 293T (293T-SPI1) cells, respectively. Dashed lines represent ΔM borders of > 2. Green, purple, and gray dots represent significantly methylated, demethylated, and insignificant probes, respectively. Numbers of DMCs are shown upper left (methylated) and bottom right (demethylated). **c** Distribution of enrichment scores for SPI1 binding motifs within ± 5000 bp of demethylated CpGs (left) and methylated CpGs (right) in SPI1-overexpressing 293T cells. X- and Y-axes show distance from probe CpG position and enrichment score, respectively. Horizontal line represents enrichment score = 0

We previously used TFBM overrepresentation analysis of DNA demethylated regions in RUNX1-overexpressing 293T cells to identify RUNX1 as a TF that induces DNA demethylation in a site-directed manner [18]. Hereafter, we refer to the TFs which induce binding site-directed DNA demethylation as “DNA-demethylating TFs” regardless of underlying mechanisms. To evaluate whether this approach can be used to detect other DNA-demethylating TFs, we investigated the TF SPI1 which has already been reported to be a DNA-demethylating TF in monocyte–osteoclast differentiation [15] (Fig. 1a).

The analysis system consisted of SPI1 overexpression and single-base resolution methylation array analysis and SPI1 binding motif overrepresentation analysis at the differentially methylated regions. Hereafter, we refer to the analysis of DNA-demethylating TFs as “demethyl-TFBM analysis.” Using Δ*M* > 2 as a cutoff, we identified 413 methylated and 1120 demethylated CpGs in SPI1-overexpressing 293T cells compared with control (293T-mock) cells, indicating a clear bias toward DNA demethylation (Fig. 1b). Subsequent TFBM overrepresentation analysis revealed that the SPI1 binding motif JASPAR_CORE; MA0080.2 (shown in Fig. 1a) is significantly overrepresented in demethylated CpG regions (1 × 10^−31^, Poisson distribution model; max enrichment score peak/Q3 + IQR = 6.01), but not in methylated regions (Fig. 1c), indicating that SPI1 determines site specificity of DNA demethylation in a binding site-directed manner. Additional ChIP-qPCR analysis for SPI1 revealed binding of SPI1 protein at demethylated regions but not at methylation unchanged regions, supporting the SPI1-mediated binding site-directed DNA demethylation (Additional file 1: Figure S1a). Thus, these results suggest that the dimethyl-TFBM analysis is applicable for the identification of other DNA-demethylating TFs.

### Identification of novel DNA-demethylating TFs

**Fig. 2:**
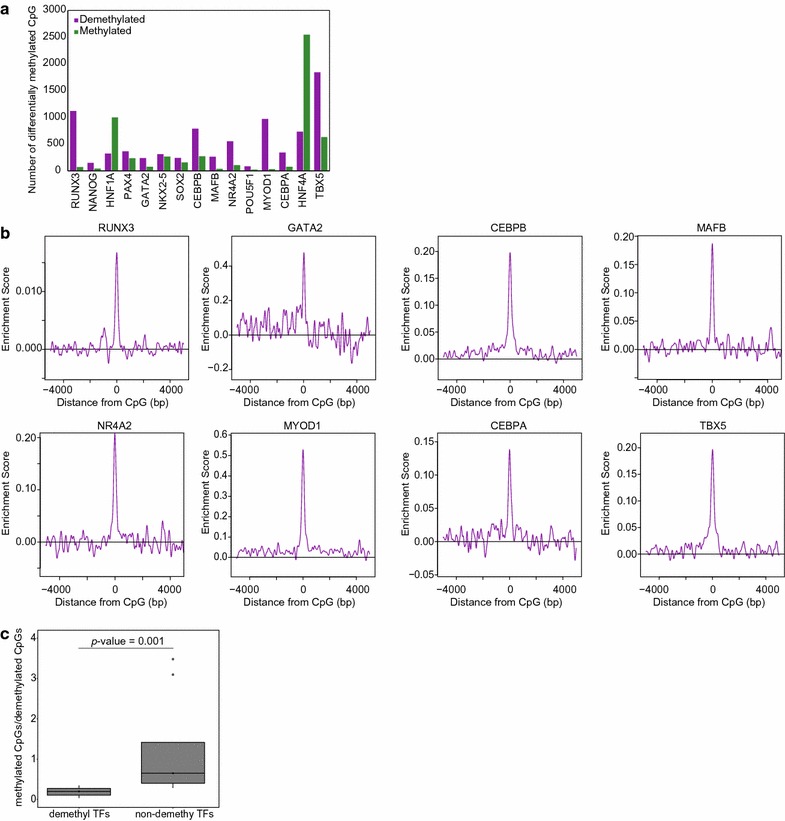
Identification of DNA-demethylating TFs. **a** Number of differentially methylated CpGs by TF overexpression. *X*- and *Y*-axes show overexpressed TFs and number of differentially methylated CpGs, respectively. Purple and green bars represent demethylated CpGs and methylated CpGs, respectively. **b** Distribution of enrichment score for TFBMs within ± 5000 bp of demethylated CpG probes in TF-overexpressing 293T cells. *X*- and *Y*-axes show distance from probe CpG position and enrichment score, respectively. Horizontal lines are enrichment score = 0. **c** Boxplot showing ratio of number of methylated and demethylated CpGs for DNA-demethylating TFs (demethyl TFs) and non-DNA-demethylating TFs (non-demethyl TFs). Medians are indicated by central black horizontal lines, upper quartiles are indicated by upper edges of the box, and lower quartiles are indicated by lower edges of the box. Maximum and minimum values are marked as lines extending from the boxes. The *p* value is shown above the plots (Wilcoxon rank sum test)

TFs involved in cellular differentiation and/or cellular function are considered to globally alter both gene expression and the epigenetic status. Therefore, to screen DNA-demethylating TFs, we applied the demethyl-TFBM analysis to 15 TFs manually selected as having important roles in cellular differentiation and/or cellular function (Table 1) [19]. The overexpression of these TFs in 293T cells was confirmed by quantitative reverse transcription (qRT)-PCR, revealing sufficiently greater expression (> 7.1-fold) compared with 293T-mock control cells (Additional file 1: Figure S1b). In subsequent methylation array analysis, we identified 24–2547 and 85–1841 methylated and demethylated CpGs, respectively, in 15 TF-overexpressing samples (Fig. 2a). TFBM overrepresentation analysis of demethylated regions revealed that the corresponding binding motifs of eight of the 15 TFs (RUNX3, GATA2, CEBPB, MAFB, NR4A2, MYOD1, CEBPA, and TBX5) were significantly overrepresented (Fig. 2b; Table 2). On the other hand, there was no overrepresentation of corresponding binding motifs in methylated regions (Additional file 1: Figure S1c). This suggested that these eight TFs induce DNA demethylation in a binding site-directed manner. Interestingly, the number of differentially methylated CpGs following the overexpression of the eight DNA-demethylating TFs was significantly biased toward demethylation compared with non-DNA-demethylating TFs (Fig. 2c), which is consistent with the results seen for SPI1 (Fig. 1b) and RUNX1 [18]. This supports a DNA demethylation role for the identified TFs.

**Table 1:** Target transcription factors selected for DNA demethylated transcription factor screening

Gene symbol	Accession	Cell type	Reference
RUNX3	NM_001031680.2	Lymphoid cells	Nat Immunol. 2015 Nov;16(11):1124–33
NANOG	NM_024865.2	Embryonic stem cells	Cell. 2003 May 30;113(5):631–42
HNF1A	NM_000545.5	Hepatocytes	Nature. 1992 Jan 30;355(6359):457–61
PAX4	NM_006193.2	Insulin-producing beta cells	Nature. 1997 Mar 27;386(6623):399–402
GATA2	NM_001145661.1	Hematopoietic cells	Nature. 1994 Sep 15;371(6494):221–6
NKX2-5	NM_004387.2	Cardiomyocytes	Genes Dev. 1995 Jul 1;9(13):1654–66
SOX2	NM_003106.2	Embryonic stem cells/neural stem cells	Genes Dev. 2003 Jan 1;17(1):126–40, Mol Cell Biol. 2004 May;24(10):4207–20
CEBPB	NM_005194	Uminal cells in the mammary gland	Stem Cells. 2010 Mar 31;28(3):535–44
MAFB	NM_005461.3	Myelomonocyte cells	Cell. 1996 Apr 5;85(1):49–60
NR4A2	NM_173173.1	T cells	Nat Commun. 2011; 2:269
POU5F1	NM_002701.4	Embryonic stem cells	Cell. 1998 Oct 30;95(3):379–91
MYOD1	NM_002478.4	Myogenic cells	Cell. 1988 Jun 3;53(5):781–93
CEBPA	NM_004364.3	Myeloid cells/adipocytes	Proc Natl Acad Sci U S A. 1997 Jan 21;94(2):569–74, Genes Dev. 1994 Jul 15;8(14):1654–63
HNF4A	NM_178850.1	Hepatocytes	Nature. 1992 Jan 30;355(6359):457–61
TBX5	NM_181486.1	Cardiomyocytes	Nat Genet. 2001 Jul;28(3):276–80

**Table 2:** Motif overrepresentation analysis

Gene	Motif ID	Motif source	Species	Poisson *p* value	Enrichment score ratio (max/outlier)	Judgment
RUNX3	RUNX3	jolma2013	Hsapiens	2.07E−33	15.48	DNA demethylating
GATA2	MA0036.1	JASPAR_CORE	Hsapiens	1.16E−29	4.29	DNA demethylating
CEBPB	MA0466.1	JASPAR_2014	Hsapiens	7.87E−250	13.80	DNA demethylating
MAFB	Mafb	jolma2013	Mmusculus	6.41E−52	11.99	DNA demethylating
NR4A2	MA0160.1	JASPAR_CORE	Mmusculus	5.89E−72	9.76	DNA demethylating
MYOD1	MA0499.1	JASPAR_2014	Mmusculus	< 1E−320	23.64	DNA demethylating
CEBPA	MA0102.2	JASPAR_CORE	Vertebrata	1.15E−89	7.96	DNA demethylating
TBX5	TBX5	jolma2013	Hsapiens	4.11E−174	13.14	DNA demethylating
NANOG	NA	SwissRegulon	NA	0.90	NA	Non-DNA demethylating
HNF1A	HNF1A	jolma2013	Hsapiens	0.12	NA	Non-DNA demethylating
PAX4	MA0068.1	JASPAR_CORE	Mmusculus	1.00	NA	Non-DNA demethylating
Nkx2-5	UP00249	UniPROBE	Mmusculus	1.00	NA	Non-DNA demethylating
SOX2	MA0143.1	JASPAR_CORE	Mmusculus	0.38	NA	Non-DNA demethylating
POU5F1	MA0142.1	JASPAR_CORE	Mmusculus	0.50	NA	Non-DNA demethylating
HNF4A	HNF4A	jolma2013	Hsapiens	0.06	NA	Non-DNA demethylating

### Functional evaluation of TF-mediated DNA demethylation regions

Because we used 293T cells as a model cells, the screening results may not reflect physiological function. To evaluate our screening results from a functional aspect, we performed gene ontology (GO) and tissue enrichment analyses of genes with demethylated CpGs at their promoter regions (demethylated genes). We first selected demethylated CpGs within regions ± 500 bp from the corresponding transcription start site of GENCODE genes (release 19). We identified 114, 47, 119, 46, 79, 135, 79, 212, and 190 promoter-associated demethylated CpGs in RUNX3-, GATA2-, CEBPB-, MAFB-, NR4A2-, MYOD1-, CEBPA-, TBX5-, and SPI1-overexpressing cells, respectively. Interestingly, the number of demethylated CpGs following the overexpression of DNA-demethylating TFs was significantly biased toward promoter regions in all identified DNA-demethylating TFs (*p* value < 10^−6^, Fisher’s exact test), suggesting that DNA-demethylating TFs preferentially demethylate promoter regions (Table 3).

**Table 3:** Number of promoter-associated demethylated CpGs

Demethylating TF	Prom-associated	Prom-nonassociated	All demethyl	% of Prom-associated	*p* value (Fisher’s exact test)
RUNX3	114	1005	1119	10.19	1.93E−10
GATA2	47	192	239	19.67	1.51E−14
CEBPB	119	670	789	15.08	2.20E−16
MAFB	46	219	265	17.36	3.00E−12
NR4A2	79	474	553	14.29	6.22E−15
MYOD1	135	836	971	13.90	2.20E−16
CEBPA	79	710	789	10.01	2.21E−07
TBX5	212	1629	1841	11.52	2.20E−16
SPI1	190	930	1120	16.96	2.20E−16
RUNX1	27	143	170	15.88	5.23E−07
All probes	26,234	459,051	485,285	5.41	

“Prom-associated” and “Prom-nonassociated” indicate the number of promoter-associated CpGs and nonpromoter-associated CpG, respectively. “All dimethyl” means total number of demethylated CpGs. *p* value represents the significance of overrepresentation of promoter-associated CpGs

**Fig. 3:**
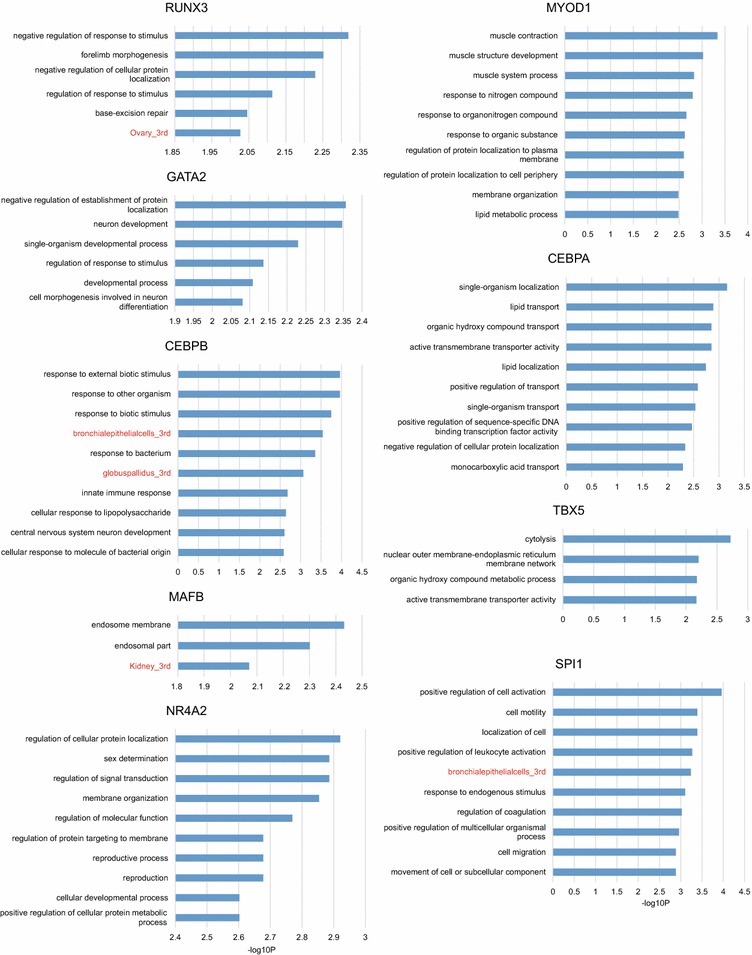
Gene ontology and specifically expressing tissue enrichment analysis. Bar plots showing enrichment of gene ontology (black labels) and specifically expressing tissues (red labels). *X*-axis represents −log_10_ *P* of Fisher’s exact test. Specifically expressed tissues were analyzed using Affymetrix GNF_U133A tissue expression data

Using the online DAVID tool (https://david.ncifcrf.gov/), we identified the top 10 enriched GOs or specifically expressing tissues at promoter-associated demethylated CpG regions for each DNA-demethylating TF (Fig. 3; full list of significantly enriched GOs or specifically expressing tissues shown in Additional file 2). The enriched GOs/specifically expressing tissues tended to be linked with corresponding cellular functions of overexpressed TFs. Notably, enriched GOs in MYOD1-overexpressing cells were clearly associated with muscle development and functions such as “muscle contraction,” “muscle structure development,” and “muscle system process.” Furthermore, the ovary was found to be enriched as a specifically expressing tissue for RUNX3-induced demethylated genes, which is consistent with the importance of RUNX3 previously shown in ovary function [20]. In addition, lipid-associated GOs such as “lipid transport” and “lipid localization” were significantly enriched in demethylated genes in CEBPA-overexpressing cells. CEBPA is a key transcription factor in adipogenesis, and the ectopic expression of CEBPA together with PPARG was previously shown to induce NIH-3T3 fibroblasts to differentiate into adipocytes [21, 22]. In contrast, no significantly enriched GOs or specifically expressing tissues associated with the overexpressed TF functions were detected at promoter-associated demethylated CpG regions for each non-DNA-demethylating TF. These results validated our screening system and indicate that DNA-demethylating TFs demethylate genomic regions associated with corresponding biological functions.

### Target CpG methylation levels of DNA-demethylating TFs

**Fig. 4:**
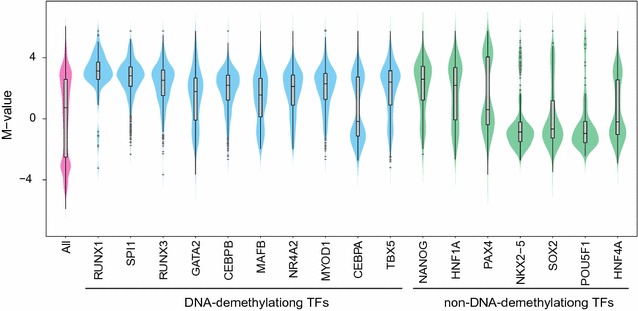
Original DNA methylation level of TF-mediated DNA demethylation targets. Violin plots showing kernel density distribution plot of original *M*-values for TF-mediated DNA demethylation targets. *M*-value distribution of all probes, of demethylated probes by DNA-demethylating TFs, and of demethylated probes by non-DNA-demethylating TFs is shown as magenta, cyan, and green, respectively. Interquartile ranges are shown as boxes in the overlaid boxplots (gray), and medians are shown as horizontal bars. Maximums and minimums are the upper and lower ends of the black line. Outliers (> Q3 + interquartile range × 1.5) are shown as gray dots

Highly methylated chromatin regions typically have low accessibility [23–26]; however, pioneer TFs can bind to closed chromatin and initiate its opening [27–31], suggesting that they have preferential epigenetic status for binding. We evaluated target CpG methylation levels among DNA-demethylating TFs and showed that they tended to be significantly biased toward hypermethylation, although average methylation levels varied (Fig. 4). Of note, CEBPA methylation levels were significantly lower than those of other DNA-demethylating TFs. On the other hand, original CpG methylation levels of DNA demethylated regions in non-DNA-demethylating TFs tended to be low with a high level of variance, suggesting that DNA demethylation by non-DNA-demethylating TFs is nonspecific and caused by multiple factors. These results indicate that DNA-demethylating TFs target highly methylated CpGs.

### Effects of TF-mediated DNA demethylation

**Fig. 5:**
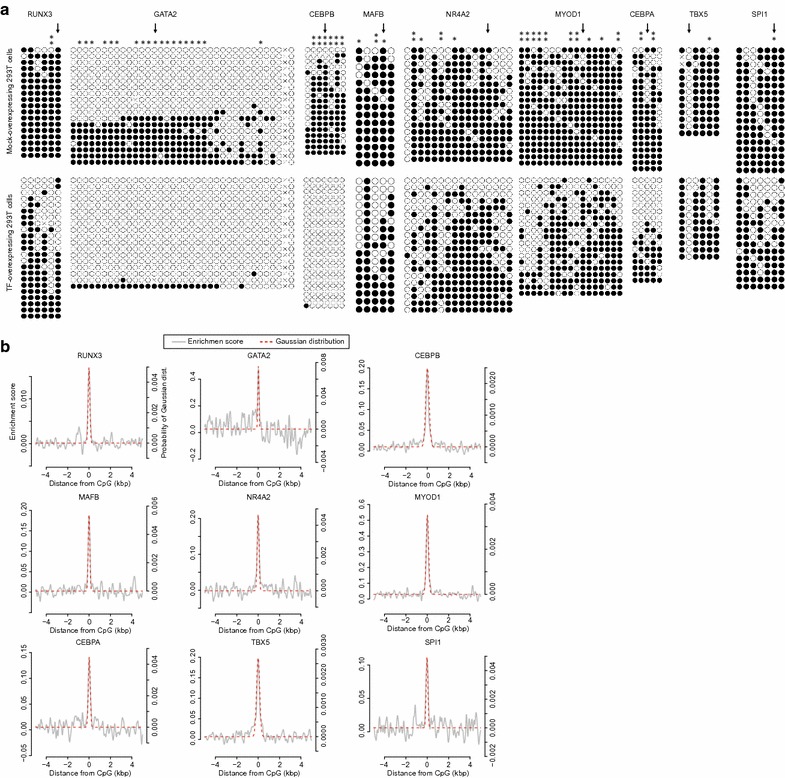
Local effect of TF-mediated DNA demethylation. **a** DNA methylation patterns of demethylated regions, selected from methylation array results, were analyzed by bisulfite-PCR sequencing. Each circle represents a cytosine of CpG. Black and white circles indicate methylated and unmethylated cytosine, respectively. Horizontal lines represent the sequencing result of each sub-clone. Arrows represent the position of demethylated CpGs identified by methylation array analysis (*p < 0.05; **p < 0.01; Fisher’s exact test). **b** Gaussian distribution model fitting of enrichment score peaks. *X*-axis shows the distance from demethylated CpGs. *Y*-axis represents the enrichment score (left) and probability of the Gaussian distribution model (right). Gray plots are enrichment scores from Figs. 1c and 2b, and red dotted plots are fitted Gaussian distributions

We previously showed that RUNX1 induced local DNA demethylation [18]. To investigate local DNA demethylation involving other DNA-demethylating TFs, we performed bisulfite-PCR sequencing of selected demethylated regions. We observed significant demethylation at the demethylated CpG probe position as well as at surrounding CpGs (Fisher’s exact test), suggesting that DNA demethylation by DNA-demethylating TFs influences an area surrounding the binding site (Fig. 5a). To estimate the demethylation range, we fitted a Gaussian distribution model to the peak of the enrichment score for the eight identified DNA-demethylating TFs and SPI1 (Fig. 5b and Additional file 3: Figure S2). We regarded ± 2*σ* of the fitted standard curve, which covers about 95% of the probability, as the TF demethylated range. 2*σ* for RUNX3, GATA2, CEBPB, MAFB, NR4A2, MYOD1, CEBPA, TBX5, and SPI1 was 161.3, 106.1, 320.8, 148.1, 122.5, 227.1, 198.2, 296.0, and 161.6, respectively, suggesting that DNA-demethylating TFs induce local DNA demethylation to a range of a few hundred base pairs.

### Proteins within the same family share demethylation targets

**Fig. 6:**
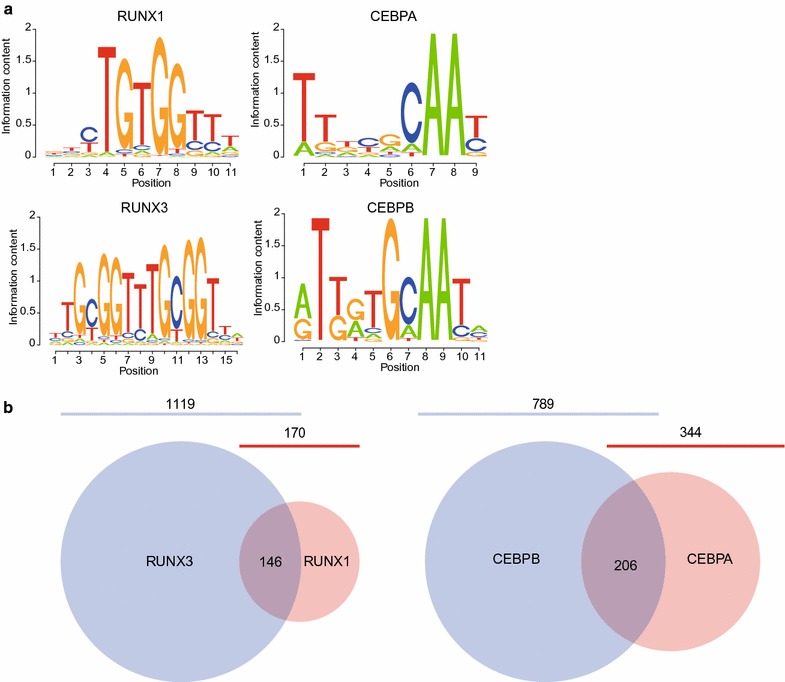
Family DNA-demethylating TFs share demethylation targets. **a** Position weight matrix-based sequence logos of TFBMs for RUNX1 (top left), RUNX3 (bottom left), CEBPA (top right), and CEBPB (bottom right). The height of each letter represents the probability of TF appearance at binding sites. **b** Overlap of demethylated CpGs between RUNX1-overexpressing (red) and RUNX3-overexpressing (blue) cells and between CEBPA-overexpressing (red) and CEBPB-overexpressing (blue) cells. The number of overlapping CpGs is shown at the intersection of each circle. The total number of demethylated CpGs is depicted as a particular circle size and is shown above the circles

The DNA-demethylating TFs identified in this study and our previous RUNX1 study [18] belong to the same family (RUNX1 and RUNX3 or CEBPA and CEBPB) and essentially recognize the same binding motifs (Fig. 6a). To evaluate the specificity of DNA demethylation caused by TFs of the same family, we examined the overlap of demethylated CpGs (Fig. 6b). Of 170 and 1119 demethylated CpGs in RUNX1- and RUNX3-overexpressing cells, 146 significantly overlapped (85.9% of RUNX1 and 13.0% of RUNX3, respectively) (*p* value = 2.2 × 10^−16^, Fisher’s exact test). Moreover, of 344 and 789 demethylated CpGs in CEBPA- and CEBPB-overexpressing cells, the overlap was 206 (60.0% of CEBPA and 26.1% of CEBPB, respectively) (*p* value = 2.2 × 10^−16^, Fisher’s exact test). These results indicate that the demethylation targets of DNA-demethylating TFs in the same family tend to coincide but do not completely overlap with each other.

## Discussion

DNA methylation dynamics are key to the understanding of development and cellular differentiation. In this study, we developed a screening system to identify TFs that regulate site-directed DNA methylation changes. The system involves the ectopic expression of TFs in 293T cells and overrepresentation analysis of the corresponding TFBM at differentially methylated regions. The use of 293T cells as model cells offers the advantages of easy handling, efficient transfection, and sufficient levels of ectopic TF expression, although 293T cells may not be suitable if the target TF is already highly expressing. Furthermore, because expression profile of binding partners and chromatin status of the model cells are also affect the results, different model cell types may lead slightly different results. Therefore, validation in a different cell type(s) strengthens of the results of our system. Thus, our system is applicable to a broad range of TFs, including TFs expressed in rare cell types and/or pathological samples that are difficult to obtain. Furthermore, the flexibility of our system enables us to analyze the relationship between abnormal DNA methylation statuses caused by mutations in DNA-demethylating TFs and the onset of diseases such as cancer.

Some TFs are involved in DNA demethylation, interacting with TET proteins [14–18]. According to FANTOM5 data (http://fantom.gsc.riken.jp/5/), expression of TETs is low in HEK293 cells (TET1: 12.65 tpm, TET2: 7.04 tpm, TET3: 1.35 tpm, GAPDH: 3259.64 tpm), but it was enough to induce RUNX1-mediated active DNA demethylation in proliferation-arrested RUNX1-overexpressing 293T cells. In addition, several reports suggest overlapping function of TETs [32, 33]. Therefore, all TET proteins may contribute to the DNA demethylation in our system.

Although our system is only applicable to TFs with a known binding motif because of TFBM overrepresentation analysis, it may be possible to predict DNA-demethylating TFs without TFBM information. For instance, we showed that DNA-demethylating TFs, but not non-DNA-demethylating TFs, significantly induced more demethylated regions than methylated ones (Fig. 2c). Therefore, the ratio between demethylated and methylated CpGs could be used as an indicator of DNA-demethylating TFs.

The ectopic expression of TFs in 293T cells may not completely reflect physiological conditions. Therefore, although the biological role of TF-mediated DNA demethylation can be predicted by GO and tissue enrichment analysis, further analysis should be performed using samples reflecting physiological conditions. One way to achieve this is a perturbation approach such as TF knockdown/out. However, the outcomes of this are difficult to distinguish as a DNA demethylation function or other TF function because one TF typically possesses multiple functions [34]. Recently, CRISPR/Cas9 or TAL-effector system-based methods for targeting DNA methylation or demethylation of specific regions have been reported [35–41]. Although these techniques have yet to be established, they are promising approaches to analyze the biological function of TF-mediated DNA methylation changes under physiological conditions.

Our findings showed that DNA-demethylating TFs induce local DNA demethylation that ranges over a few hundred base pairs. Typical differential methylation analysis adopts a sliding window of around 1 kb across the genome [42], but our results suggest that a broader window size may miss DNA-demethylating TF-mediated DNA demethylation. Indeed, during cell reprogramming, local DNA demethylation at key TF binding sites at early time points would not be detected by broader differential methylation analysis [43]. Because we estimated the effect of the DNA-demethylating TFs based on the methylation array data, validation experiments will be needed to precisely determine the demethylation range.

The TFs RUNX1 and RUNX3, and CEBPA and CEBPB, respectively, share a large number of demethylation targets (Fig. 6b), which is consistent with the functional redundancy between RUNX1 and RUNX3 [44] or between CEBPA and CEBPB [45]. However, the expression spectra differ among the same family of TFs [46]. RUNX1 is most highly expressed in CD14+ monocytes, while the highest RUNX3 expression is detected in CD8+ T cells and natural killer cells [47]. Similarly, CEBPA is most highly expressed in mature adipocytes, while CEBPB is mainly expressed in myeloid lineage cells [47]. Furthermore, although CEBPA and CEBPB proteins generally occupy the same chromatin regions, they show distinct quantitatively divergent temporal patterns because of their different association partners during liver regeneration [48]. Therefore, as well as TFBM, expression patterns and association partners may also determine the spatiotemporal regulation of DNA demethylation dynamics.

Our system can theoretically identify TFs involved in both DNA methylation and demethylation. Nonetheless, it was surprising that we identified only eight out of 15 TFs as DNA-demethylating TFs and that we found no TFs involved in DNA methylation. Because the expression of de novo DNMTs (DNMT3A and DNMT3B) is low in HEK293 cells (4.03 and 9.12 tpm, respectively, according to FANTOM5 data), the activity of the enzyme may not be sufficient. Therefore, compensating the de novo methylation activity, co-overexpression of de novo DNMT with the TF may enable our system to identify the TFs involved in DNA methylation. On the other hand, we have reported that enrichment of TF binding motifs is biased toward demethylated regions rather than methylated regions in hematopoietic differentiation [18]. Therefore, the results of our study also likely reflected the predominance of DNA demethylation, although the selection of these 15 TFs was manual curation based on literature information, which may have a bias. Thus, TFs involved in DNA demethylation represent a novel major functional subclass, which could be further explored by scaling up our analysis.

Under physiological conditions, DNA binding factors such as TFs have been shown to locally influence DNA methylation [49]. We previously identified several significantly overrepresented TFBMs including the RUNX1 motif in DNA demethylated regions of multiple hematopoietic differentiation pathways [18]. This suggested that many TFs are involved in site-specific DNA methylation changes. Large-scale research projects such as the Roadmap Epigenetics Project and the Blueprint project have made genome-wide methylome data publicly available [50, 51]. These data can be used to systematically identify overrepresented TFBM in differentially methylated regions, which will reveal a more global view of the TF involvement in DNA methylation changes. However, because the same or similar TFBMs are shared by a set of TFs [52], further follow-up experiments are necessary to fully identify corresponding DNA-demethylating TFs. Our approach can be used to identify such TFs because of the one–one relationship between overrepresented motifs and overexpressed TFs.

## Conclusions

Our results emphasize the usefulness of the developed screening system for the identification of DNA-demethylating TFs. Furthermore, we used the system to identify eight novel DNA-demethylating TFs that are likely to play important roles in biological processes.

## Methods

### Cell culture

293T cells were acquired from the RIKEN Bio Resource Center (BRC) and were cultured in Dulbecco’s modified Eagle’s medium (Wako Pure Chemical Industries, Ltd, Osaka, Japan) supplemented with 10% fetal bovine serum and penicillin/streptomycin (100 U/mL, 100 µg/mL; Thermo Fisher Scientific Inc., Waltham, MA, USA).

### Ectopic expression of target TF genes

Target TF genes were sub-cloned into the CSII-EF-RfA-IRES2-puro vector [18] by the Gateway LR recombination technique. Lentivirus vector production was performed as previously described [53] and then used to infect 293T cells with a multiplicity of infection of 1. Puromycin selection at 2 μg/mL was carried out for 1 week. qRT-PCR was performed as previously described [53] to confirm mRNA expression levels. The primer set for each target is shown in Additional file 4.

### Methylation array analysis

The Infinium™ HumanMethylation450 BeadChip (Illumina Inc., San Diego, CA, USA) was used to profile DNA methylation as previously described [18]. Briefly, genomic DNA was isolated using a NucleoSpin^®^ Tissue Kit (Macherey–Nagel GmbH & Co., Düren, Germany) followed by bisulfite C–T conversion using the EZ DNA Methylation-Gold™ Kit (Zymo Research Corp., Irvine, CA, USA) according to the manufacturer’s instructions. Genomic DNA was then subjected to methylation array analysis according to the manufacturer’s instructions. Normalization and the *M*-value, a statistical metric for log-scale methylation levels, were computed using the lumi Bioconductor package [54]. An *M*-value difference (∆*M*) cutoff of ≥ 2 was used to identify differentially methylated CpGs.

### Screening of site-directed DNA demethylation-inducing transcription factors

We performed TFBM overrepresentation analysis as previously described [18]. Briefly, sequences located ± 5 kbp from the methylated or demethylated probe positions and the same number of randomly selected probes were extracted from version hg19 of the human genome sequence. TFBM identification was performed using the matchPWM command of the Biostrings package, and the MotifDb database package of the Bioconductor or SwissRegulon weight matrix database (http://swissregulon.unibas.ch/data/hg19_f5/hg19_weight_matrices_v2) was used for the overrepresentation analysis. Enrichment scores were calculated using the following formula:

$${\text{Enrichment}}\,{\text{score}} = \frac{{\left( {\mathop \sum \nolimits_{i}^{n} K\left( {\frac{{x - x_{ti} }}{h}} \right) - \mathop \sum \nolimits_{j}^{n} K\left( {\frac{{x - x_{cj} }}{h}} \right) } \right) }}{n}$$

Here, *i* and *j* are identified motifs in differentially methylated regions and randomly selected controls, respectively, and *χ*_*t*_ and *χ*_*c*_ donate motif positions in differentially methylated regions and randomly selected controls, respectively. The smoothing parameter h was set at 50, and *K* was calculated as a Gaussian kernel function as follows:

$$K = \frac{1}{{\sqrt {2\pi } }} \cdot {\text{e}}^{{ - \frac{1}{2}x^{2} }}$$

To judge whether the TF is DNA-demethylating TF, first, statistical overrepresentation of the motif in differentially methylated CpG regions compared with randomly selected CpG regions was computed (Poisson distribution model, *p* value < 0.00001). Next, we further selected overrepresented motifs with a maximum enrichment score at the methylated/demethylated probes of Q3 + IQR × 3 in a ± 5-kbp region.

### ChIP-qPCR analysis

Chromatin immunoprecipitation (ChIP) assays were performed as described previously [55] using anti-SPI1 (Cell Signaling Technology, Danvers, MA, USA; cat no. 2258) antibody. ChIPed DNA was subjected to real-time PCR. Fold enrichment is calculated as the ration of ChIPed DNA to IgG control. The primers are shown in Additional file 5.

### Bisulfite sequencing

Genomic DNA was subjected to bisulfite C–T conversion, as described above. The target genomic region was amplified from genomic DNA using EpiTaq™ HS (Takara Bio Inc., Shiga, Japan) using primers shown in Additional file 6. The PCR products were cloned into the pTA2 plasmid using a TArget™ Clone Kit (Toyobo Co., Ltd., Osaka, Japan) and sequenced using BigDye^®^ Ver3.1 (Thermo Fisher Scientific Inc.) with the 3730 × 1 DNA Analyzer (Thermo Fisher Scientific Inc.). Twenty-four clones were sequenced for each target.

### Standard curve fitting

Noise was defined as a range between the minimum and maximum enrichment scores of the ± 5-kbp region except for the central peak region. The signal was defined as the central peak, excluding the noise range. A Gaussian distribution model was then fitted to the signal, and 2*σ* was calculated based on the fitted Gaussian distribution model.

## Additional files

**Additional file 1: Figure S1.** TF overexpression and TFBM overrepresentation in methylated regions. (**a**) ChIP-seq analysis for PU.1 binding. Fold enrichment is the ration of ChIPed DNA and IgG control. Error bars represent SD. The experiments were performed in 2 biological replicates. (**b**) Fold change of overexpressed TFs. X- and Y-axes show overexpressed TFs and fold change of the expression compared with mock control (log_2_ scale), respectively. Mean and standard deviation (error bars) are shown. The experiment was performed in triplicate. (**c**) Distribution of enrichment scores for TF binding motifs within ± 5000 bp of differentially methylated CpGs in TF-overexpressing 293T cells. X- and Y-axes show distance from probe CpG position and enrichment score, respectively. Horizontal lines are enrichment score = 0.

**Additional file 2.** Full list of gene ontology and specifically expressing tissue enrichment analysis. *p* values were computed by Fisher’s exact test.

**Additional file 3: Figure S2.** Schematic workflow of Gaussian distribution model fitting. The signal is detected as the peak closest to the CpG position (pink), and the noise is the range of nonsignal peaks (gray). The Gaussian distribution model was fitted to the signal peak, and ± 2σ was computed.

**Additional file 4.** Primer list for qRT-PCR.

**Additional file 5.** Primer list for ChIP-qPCR.

**Additional file 6.** Primer list for bisulfite-PCR sequencing.

## Abbreviations

TFs: transcription factors
TFBM: TF binding motif
293T-mock: mock control-overexpressing 293T cells
GO: gene ontology
tpm: tag per million
