# Supplementary figures and images for: A screening system to identify transcription factors that induce binding site-directed DNA demethylation
Source: Epigenetics Chromatin. 2017 Dec 8;10:60. doi: 10.1186/s13072-017-0169-6 (PMC5723091; doi:10.1186/s13072-017-0169-6)

# Figure S1

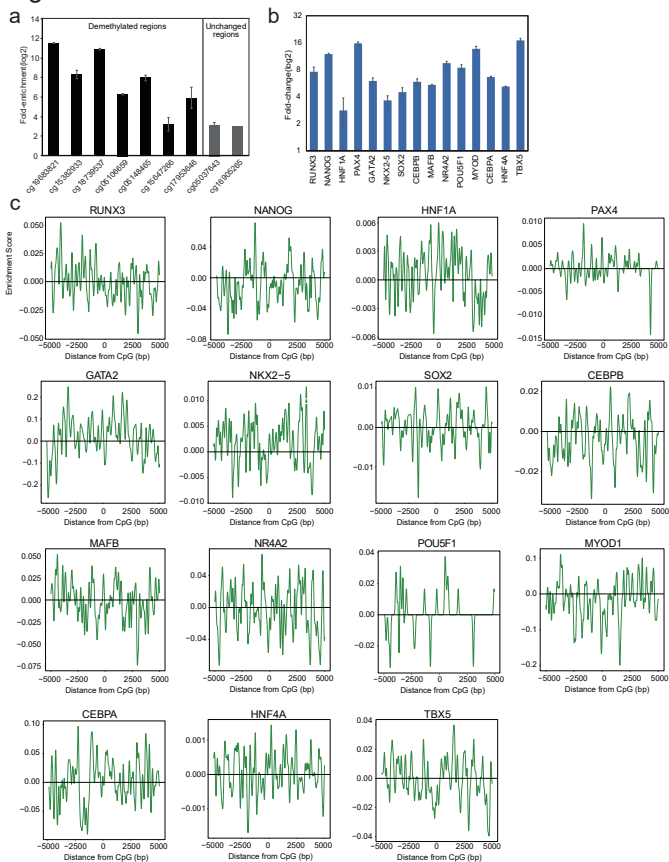

Supplement: Supplementary file 1 — Additional file 1: Figure S1. TF overexpression and TFBM overrepresentation in methylated regions. (a) ChIP-seq analysis for PU.1 binding. Fold enrichment is the ration of ChIPed DNA and IgG control. Error bars represent SD. The experiments were performed in 2 biological replicates. (b) Fold change of overexpressed TFs. X- and Y-axes show overexpressed TFs and fold change of the expression compared with mock control (log2 scale), respectively. Mean and standard deviation (error bars) are shown. The experiment was performed in triplicate. (c) Distribution of enrichment scores for TF binding motifs within ± 5000 bp of differentially methylated CpGs in TF-overexpressing 293T cells. X- and Y-axes show distance from probe CpG position and enrichment score, respectively. Horizontal lines are enrichment score = 0. [file 13072_2017_169_MOESM1_ESM.pdf]

Figure S2

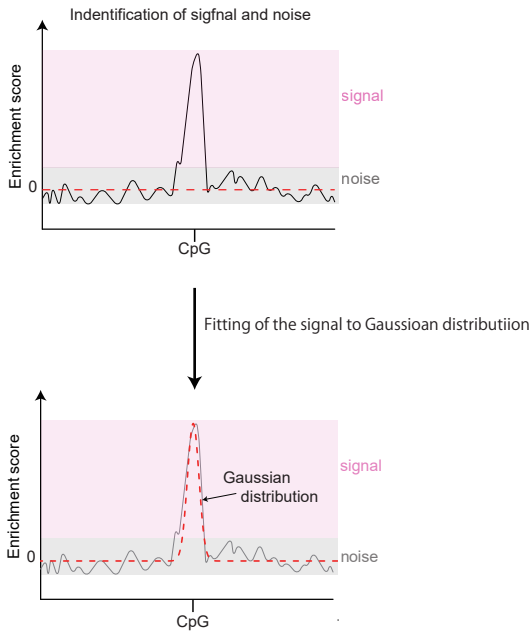

Supplement: Supplementary file 3 — Additional file 3: Figure S2. Schematic workflow of Gaussian distribution model fitting. The signal is detected as the peak closest to the CpG position (pink), and the noise is the range of nonsignal peaks (gray). The Gaussian distribution model was fitted to the signal peak, and ± 2σ was computed. [file 13072_2017_169_MOESM3_ESM.pdf]
